# The Periodontal–Cardiovascular Disease Association: Molecular Mechanisms and Clinical Implications

**DOI:** 10.3390/ijms26167710

**Published:** 2025-08-09

**Authors:** Elisabetta Ferrara, Alessandro D’Albenzio, Jessica Bassignani, Isabella Di Tanna, Giovanna Murmura, Giuseppe Balice

**Affiliations:** 1 Department of Human Sciences, Law, and Economics, Telematic University “Leonardo Da Vinci”—UNIDAV, Torrevecchia Teatina, 66100 Chieti, Italy; 2 Complex Operative Unit of Pathological Addiction, Addiction Service, ASL2 Abruzzo, 66100 Chieti, Italy; alessandro.dalbenzio@asl2abruzzo.it; 3 European Institute of Medical Studies (EIMS), STJ 3150 San Ġiljan, Malta; jessica.bassignani@eims.edu.mt; 4 Independent Researcher, 65015 Pescara, Italy; isabella841@hotmail.it; 5 Department of Innovative Technologies in Medicine & Dentistry, University “G. d’Annunzio” Chieti-Pescara, Via dei Vestini 31, 66100 Chieti, Italy; giovanna.murmura@unich.it (G.M.); giuseppe.balice@phd.unich.it (G.B.)

**Keywords:** periodontal disease, cardiovascular disease, inflammation, NLRP3 inflammasome, oxidative stress, epigenetic modification, endothelial dysfunction

## Abstract

The relationship between periodontitis and cardiovascular diseases (CVDs) extends beyond epidemiological associations, as demonstrated by meta-analyses showing a significantly increased risk for coronary heart disease development. At the core of this association lies systemic inflammation, where periodontal pathogens initiate cascades of pro-inflammatory cytokines. This inflammatory response manifests through substantial elevations in interleukin-1 beta (IL-1β), tumor necrosis factor-α (TNF-α), and interleukin-6 (IL-6) in periodontitis patients. Oxidative stress plays a crucial role, with Nicotinamide Adenine Dinucleotide Phosphate (NADPH) Oxidase 2 (NOX2) activation leading to markedly increased superoxide production compared to healthy controls. The peroxynitrite formed via NO–superoxide interaction accumulates in affected vascular tissues, substantially reducing nitric oxide (NO) bioavailability. Molecular mimicry mechanisms are evidenced by *P. gingivalis* heat shock protein sharing significant sequence homology with human HSP60, triggering autoimmune responses that affect cardiovascular tissues. Epigenetic modifications show specific alterations, with Nrf2 target gene expression substantially downregulated in chronic periodontal inflammation, particularly affecting heme oxygenase-1 (HO-1) and NAD(P)H:Quinone Oxidoreductase 1 (NQO1) expression. These molecular pathways create a complex network of interactions that fundamentally link periodontal and cardiovascular pathologies.

## 1. Introduction

The periodontal–cardiovascular axis has emerged as a critical focus in medical research since the late twentieth century. The recognition that periodontitis transcends its classification as a localized oral condition to manifest significant systemic effects has transformed contemporary understanding of oral–systemic interactions [1,2]. The implications of this relationship acquire particular relevance in light of cardiovascular diseases (CVDs) remaining the leading cause of global mortality, accounting for 17.9 million deaths annually and representing 32% of all global deaths [3]. Analysis of 29 studies encompassing over 57,000 participants has established that periodontitis confers a 1.14 to 2.2-fold increased risk for coronary heart disease development [4]. These findings receive further validation through longitudinal investigation of 1400 participants over 24 months, demonstrating hazard ratios of 1.1 to 2.0 for CVDs, following adjustment for traditional risk factors [5]. Contemporary epidemiological evidence provides robust validation for the association between periodontal disease and CVDs, with systemic inflammation serving as the common underlying denominator. Recent findings from Matsuda et al. [6] demonstrate that in systemically healthy individuals, periodontitis significantly correlates with elevated systemic inflammatory markers. Their multicenter investigation of 153 participants revealed a significant association between periodontal inflamed surface area and serum IL-6 levels at baseline (β = 0.191, *p* = 0.021). Notably, their research established that periodontal treatment effectively reduced serum IL-6 levels independent of periodontal pathogen infection, providing mechanistic insights into how periodontal inflammation may predispose to CVDs development [6]. The translational significance of these epidemiological associations is further substantiated by Lyu et al. [7] in a comprehensive systematic review and meta-analysis of 14 randomized controlled trials encompassing 491 periodontitis patients. Their analysis demonstrated that periodontal treatment significantly improved endothelial function as measured by flow-mediated dilation in both short-term (≤3 months: WMD = −3.78, 95% CI = [−5.49, −2.07], *p* < 0.0001) and long-term (6 months) follow-up, with particularly pronounced effects in patients with severe periodontitis. To elucidate the biological mechanisms underlying these epidemiological and interventional findings, it is essential to examine the specific molecular pathways involved. These pathogenic processes encompass inflammatory cascades, oxidative stress mechanisms, and direct bacterial effects, which collectively establish the mechanistic links between oral inflammation and systemic vascular pathology [8]. Systemic inflammation emerges as a central mediator, with periodontitis inducing chronic low-grade inflammation marked by elevated levels of pro-inflammatory cytokines—namely interleukin-1 beta (IL-1β), tumor necrosis factor-alpha (TNF-α), and interleukin-6 (IL-6)—which exacerbate atherosclerosis and contribute to vascular dysfunction [9]. Oxidative stress represents another critical pathway in this relationship. Periodontal inflammation stimulates the release of reactive oxygen species (ROS) from activated neutrophils [10,11], resulting in oxidative damage to lipids, proteins, and DNA and favoring CVDs. This oxidative burden promotes endothelial dysfunction and accelerates the progression of atherosclerosis in both local and systemic vascular territories [9]. Additionally, molecular mimicry has been implicated as a significant mechanistic interface, characterized by structural homology between bacterial and host proteins. This phenomenon is particularly evident in the cross-reactivity between *Porphyromonas gingivalis* antigens and host proteins involved in atherosclerosis [12]. Direct bacterial invasion further contributes to cardiovascular pathology, with periodontal bacterial DNA detected within atheromatous plaques, promoting localized inflammation and immune activation [13]. Among these mimicry mechanisms, *P. gingivalis* heat shock protein GroEL shares approximately 60% sequence homology with human HSP60, particularly in immunodominant epitopes. This structural similarity elicits the production of autoantibodies capable of targeting both bacterial and host cardiovascular tissues, thereby potentially contributing to atherosclerotic lesion development [14,15]. Recent advances in molecular biology have revealed further mechanistic insights. Epigenetic modifications have emerged as important contributors, with inflammation induced by periodontitis leading to heritable changes in gene expression. These alterations affect immune and inflammatory pathways, with specific DNA methylation signatures identified in both periodontal and CVDs [15,16]. Moreover, dysregulated expression of microRNAs—particularly miR-146a and miR-155—suggests shared regulatory networks across both conditions [17]. Technological progress has enabled deeper exploration of these mechanisms. Extracellular vesicle signaling has been recognized as a key mediator, with periodontal pathogens releasing vesicles that carry bioactive molecules capable of influencing distant cardiovascular tissues [7,15,18,19]. Furthermore, periodontal inflammation induces systemic metabolic dysregulation, altering lipid metabolism and glucose homeostasis, which may accelerate the onset and progression of atherosclerosis [20]. Collectively, these molecular insights hold significant clinical relevance, supporting the development of targeted therapeutic strategies such as anti-inflammatory agents, antioxidant therapies, and antimicrobial interventions. This review has highlighted the multifactorial molecular mechanisms linking periodontal and CVDs, with a particular focus on inflammatory mediators, oxidative stress, and epigenetic regulation. While numerous reviews have examined the periodontal–cardiovascular connection, this review provides unique contributions: (1) a quantitative analysis of molecular changes with specific fold-changes and concentrations, (2) the integration of three major pathways (NLRP3, oxidative stress, ADMA) showing their convergence points, (3) the incorporation of recent findings on epigenetic regulation and extracellular vesicle signaling, and (4) the identification of specific molecular targets for therapeutic intervention.

## 2. Oral Microbiome Dysbiosis and Cardiovascular Pathogenesis

To understand these cardiovascular sequelae at the molecular level, it is essential to first examine the oral microbiome alterations that initiate this pathological cascade. Three major aspects characterize the oral microbiome’s role in cardiovascular pathogenesis: polymicrobial synergy, bacterial translocation mechanisms, and metabolic perturbations. Contemporary metagenomic analyses, facilitated by advanced sequencing technologies, have revolutionized our understanding of the oral microbiome’s role in cardiovascular pathophysiology [21,22,23]. This sophisticated ecological network exhibits complex regulatory mechanisms characterized by polymicrobial synergy and dysbiosis, wherein transitions from health-associated species to pathobionts occur through precise alterations in metabolic networks and virulence gene expression patterns [24].

*P. gingivalis* demonstrates molecular sophistication through its gingipain proteases—arginine-specific (RgpA/B) and lysine-specific (Kgp) variants—which exhibit environmentally modulated catalytic activity [25]. These cysteine proteases initiate signaling cascades through PAR receptor activation, particularly PAR1 and PAR2, modulating host inflammatory responses [26,27]. The molecular architecture of *P. gingivalis* lipopolysaccharide exhibits remarkable structural plasticity, characterized by dynamic transitions between penta- and tetra-acylated lipid A configurations, facilitating sophisticated immune modulation [28]. This structural dynamism directly influences TLR4/MD2 complex assembly and subsequent MyD88-dependent signaling cascades [8].

Bacterial translocation proceeds through precisely orchestrated molecular mechanisms: *P. gingivalis* activates MAPK signaling through site-specific dual phosphorylation of p38 MAPK (Thr180/Tyr182), initiating a sophisticated cascade encompassing MAP3K and MAP2K activation [29]. This signaling axis culminates in the expression of matrix metalloproteinases MMP-8 and MMP-9, which exhibit specific catalytic activity against type I collagen and basement membrane constituents [26].

Bacterial virulence factors facilitate systemic dissemination through sophisticated delivery systems. *P. gingivalis*-derived outer membrane vesicles (OMVs), ranging from 20 to 250 nm, encapsulate proteolytic enzymes and adhesins optimized for stable cargo transport [17,18].

The metabolic perturbations induced by periodontal pathogens involve intricate molecular cascades, including modulation of ABCA1/ABCG1 transporters through LXRα pathway interference. Additionally, insulin signaling disruption occurs through specific post-translational modifications of IRS-1 at defined serine residues (307, 636, 639) [19].

*A. actinomycetemcomitans* exhibits precisely regulated interactions with the coagulation cascade through its sophisticated virulence factors, notably leukotoxin and cytolethal distending toxin. These molecular effectors demonstrate calcium-dependent interactions with coagulation Factor X, requiring specific physiological calcium concentrations (2.5–5 mM) for optimal catalytic activity [30]. These molecular interactions between diverse periodontal pathogens and host systems—from *P. gingivalis* gingipains to *A. actinomycetemcomitans* toxins—collectively demonstrate how the connection between oral health and cardiovascular disease has evolved from correlation to mechanistic understanding, with polymicrobial periodontal infections showing significantly greater pathogenic effects than single bacterial species [8,31]. Red complex bacteria—*Porphyromonas gingivalis*, *Treponema denticola*, and *Tannerella forsythia*—demonstrate synergistic interactions that amplify inflammatory responses, with patients carrying high antibody responses to these pathogens showing hazard ratios of 3.01–3.11 for cardiovascular events when combined with elevated inflammatory markers [32]. These bacteria utilize distinct molecular mechanisms: *P. gingivalis* employs gingipain proteases and LPS variations, A. actinomycetemcomitans produces leukotoxin and cytolethal distending toxin, *F. nucleatum* uses FadA adhesin for vascular interaction, and *T. denticola* contributes through its periplasmic flagella and synergistic biofilm formation [28,33,34].

The synergistic effects of periodontal bacteria extend far beyond simple additive responses. In controlled experimental settings, dual-species biofilms of *P. gingivalis* and *T. denticola* demonstrate significantly enhanced biomass production compared to individual species biofilms [35]. Previous research has shown that both bacterial species experience substantial increases in biovolume when grown together, with *T. denticola* showing particularly marked enhancement in polymicrobial microcolony formation [36]. This bacterial cooperation depends on specific mechanisms, notably *T. denticola* motility. Studies using flagellar hook protein deletion mutants (ΔflgE) reveal dramatic reductions in dual-species biofilm formation, highlighting the critical importance of bacterial movement in establishing these synergistic communities [35]. *T. denticola*’s periplasmic flagella enable movement through viscous environments, potentially creating pores in the biofilm matrix that enhance nutrient flow and waste removal, thereby sustaining higher biofilm biomass [36]. The inflammatory consequences of these polymicrobial interactions prove equally striking. Mixed infections with *P. gingivalis* plus either *T. forsythia* or *T. denticola* induce synergistic IL-6 production in macrophages, an effect absent with other cytokines like TNF-α or IL-1β [37]. This selective inflammatory enhancement requires *P. gingivalis* gingipains, proteolytic enzymes that facilitate bacterial cooperation. In animal models, polymicrobial infections significantly accelerate atherosclerotic plaque formation (*p* < 0.05) while increasing oxidized LDL and decreasing nitric oxide levels (*p* < 0.01), markers of endothelial dysfunction and cardiovascular risk [31].

Large-scale clinical studies provide compelling evidence for the oral–systemic connection. The ORIGINS study, examining 787 healthy individuals with over 1100 subgingival plaque samples, identified a microbial indicator of periodontitis (MIP) based on the Treponema:Corynebacterium ratio that correlates significantly with cardiovascular risk factors [38]. Remarkably, this bacterial ratio shows positive correlations with systolic blood pressure (*p* < 0.05), diastolic blood pressure (*p* < 0.05), fasting insulin (*p* < 0.05), and insulin resistance (HOMA-IR, *p* < 0.05). The classification accuracy reaches 83% (0.83 ± 0.04) using only these two bacterial genera, while the full dataset achieved 88% accuracy.

Inflammatory marker elevations in periodontitis patients demonstrate clear dose–response relationships. In a study of 189 subjects, severe periodontitis patients showed gingival crevicular fluid IL-1β levels of 61.04 ± 41.41 pg/mL, significantly higher than controls [39]. These elevated local inflammatory markers translate into systemic effects that prove equally pronounced—generalized periodontitis patients exhibit median C-reactive protein levels of 1.45 mg/L compared to 0.90 mg/L in healthy controls (*p* = 0.030), with 52% testing positive for elevated IL-6 versus 26% of controls (*p* = 0.008) [40]. The doubling of IL-6 positivity and significant CRP elevation illustrate how local periodontal inflammation manifests systemically, as these inflammatory changes correlate with increased leukocyte counts, primarily driven by neutrophil elevation (*p* = 0.001).

The presence of periodontal bacteria in atherosclerotic plaques provides direct evidence for bacterial involvement in cardiovascular pathology. A meta-analysis examining multiple studies identified oral bacteria in atherosclerotic plaques, with *P. gingivalis* showing a prevalence of approximately 40% in coronary atheromatous plaques from patients with myocardial infarction [13]. Detection methods vary in sensitivity—16S rRNA gene sequencing dominates recent studies, while earlier work relied on PCR and immunohistochemistry.

Specific detection rates reveal the prevalence of these red complex species in vascular lesions. Mahendra et al. found *T. denticola* in 51% of coronary plaques from periodontitis patients, *P. gingivalis* in 45.1%, and *T. forsythia* in 31.4% [41]. The polymicrobial nature of these infections proves critical—bacterial DNA appears in 95% of vascular biopsies, with most containing multiple species [42]. A positive correlation exists between the number of bacterial species detected in aortas and total atherosclerotic plaque area (R^2^ = 0.192, *p* < 0.005).

These bacteria do not merely colonize plaques passively. They form biofilm deposits within carotid arterial plaques, with 93% located proximal to the internal elastic lamina [33]. In experimental models, viable *T. denticola* and *Fusobacterium nucleatum* survive in cardiovascular tissues for at least 14 days post-inoculation, demonstrating persistence in the vascular environment [31]. Gene expression analysis reveals that polymicrobial infection alters 6 of 84 atherosclerosis-related genes by more than 3-fold, including upregulation of neuropeptide Y (promoting atherosclerosis) and downregulation of apolipoprotein genes crucial for lipid transport [43]. The mechanisms underlying bacterial synergy provide insights for intervention strategies. *P. gingivalis* gingipains prove essential for synergistic biofilm formation and inflammatory responses, with these proteolytic enzymes facilitating interactions with both *T. denticola* and *T. forsythia* [34]. *T. forsythia* growth specifically depends on *P. gingivalis* Lys-gingipain, while *T. denticola* periplasmic flagella enable motility-dependent biofilm cooperation [34]. This bacterial interdependence suggests that targeting key species or their virulence factors could disrupt entire pathogenic communities. These sophisticated molecular pathways—from bacterial adhesion to protease activity to vesicle-mediated communication—demonstrate the complexity of oral microbiome interactions with host systems. Such mechanisms extend beyond individual bacterial species to create synergistic pathogenic communities.

The inflammatory cascade triggered by polymicrobial infections involves multiple pathways. Bacterial components activate pattern recognition receptors, initiating NF-κB signaling and NLRP3 inflammasome assembly, which amplifies the inflammatory response through IL-1β and IL-18 production [44,45,46]. All these aspects also stimulate the oxidative stress mechanisms, which demonstrate precise molecular choreography: NOX2 activation in periodontal tissues initiates a cascade of reactive oxygen species generation, beginning with superoxide (O_2_•^−^) production and its subsequent dismutation to hydrogen peroxide (H_2_O_2_) via superoxide dismutase. The enzymatic activity of myeloperoxidase further generates hypochlorous acid (HOCl), contributing to tissue damage through specific oxidative modifications [9]. Additionally, the formation of peroxynitrite (ONOO−) through NO–superoxide interaction results in specific protein modifications and cellular dysfunction.

These sophisticated molecular pathways suggest multiple therapeutic interventions of precisely targeted nature:

- Selective inhibition of gingipain activity through structure-based protease inhibitor design.
- Targeted modulation of inflammatory cascades, with particular emphasis on NLRP3 inflammasome assembly regulation.
- Specific antioxidant interventions directed at ROS-generating enzymatic systems.
- Precise regulation of vesicle-mediated bacterial communication networks [47].

While the pathogenic mechanisms of periodontal bacteria have been extensively characterized, it is important to note that the oral microbiome also harbors beneficial species with protective properties. In contrast to these pathogenic mechanisms, beneficial oral bacteria such as *S. salivarius* demonstrate protective molecular sophistication. *S. salivarius* is exemplified by its production of structurally distinct bacteriocins: salivaricin A (22 amino acids) and salivaricin B (25 amino acids), characterized by post-translationally modified lanthionine bridges [48]. These lantibiotics demonstrate precise molecular targeting of the IKK complex, specifically inhibiting its assembly and subsequent IκB-α phosphorylation. This mechanistic intervention results in quantifiable attenuation of inflammatory adhesion molecule expression. This dual nature of the oral microbiome—harboring both pathogenic and protective species—underscores the importance of maintaining microbial balance for cardiovascular health. The intricate interplay between these molecular mechanisms underscores the necessity for sophisticated, multi-targeted therapeutic approaches in addressing the complex pathophysiology of periodontal–CVDs manifestations [8].

## 3. Molecular Mechanisms of *Porphyromonas gingivalis*-Induced Endothelial Dysfunction

*P. gingivalis*, a key periodontal pathogen, employs multiple sophisticated molecular mechanisms to compromise endothelial cell function, establishing a critical link between periodontal and CVDs [27]. *P. gingivalis* employs sophisticated molecular mechanisms to compromise endothelial cell function. The initial interaction involves FimA, the major fimbrial protein, which specifically binds to α5β1 integrin on endothelial cells. This binding triggers a precise phosphorylation cascade, beginning with focal adhesion kinase (FAK) phosphorylation at Tyr397, followed by paxillin phosphorylation at Tyr31 and Tyr118 [47]. This cascade reorganizes the actin cytoskeleton, facilitating bacterial internalization through lipid rafts.

Once internalized, the bacterium ensures its intracellular survival through the secretion of SerB, a serine phosphatase that specifically targets Rab7 GTPase, preventing endosome-lysosome fusion. This sophisticated evasion strategy allows *P. gingivalis* to persist within early endosomes, effectively avoiding lysosomal degradation [49].

Beyond these direct cellular effects, the pathogen also modulates endothelial function through epigenetic mechanisms. *P. gingivalis* upregulates DNMT1 expression, which results in increased DNA methylation at specific CpG sites in the eNOS promoter. This epigenetic modification reduces Sp1 binding and consequently decreases eNOS transcription, ultimately impairing nitric oxide production [15].

These three coordinated mechanisms—adhesion and invasion, intracellular survival, and epigenetic regulation—work synergistically to establish persistent endothelial dysfunction. Central to *P. gingivalis* pathogenicity are its gingipain proteases, which orchestrate additional pathological processes. Gingipains directly compromise vascular barrier integrity through RgpA-mediated proteolysis of VE-cadherin at the specific Arg689-Val690 site, releasing a 90 kDa soluble fragment and disrupting adherens junctions [27]. Additionally, both RgpA and RgpB gingipains modulate coagulation processes by activating PAR-2 through N-terminal cleavage, which increases intracellular calcium and activates PKC, ultimately enhancing AP-1 binding to the TF promoter and upregulating tissue factor expression [25]. Furthermore, these proteases achieve systemic dissemination through sophisticated mechanisms—gingipains secreted within outer membrane vesicles (OMVs) resist host proteases, allowing them to cross the blood–brain barrier and accumulate in distant organs [18].

Beyond these gingipain-mediated effects, lipopolysaccharide-mediated TLR4 activation initiates the inflammatory response pathway, triggering a signaling cascade through MyD88, IRAK1, and TRAF6. This leads to NF-κB phosphorylation and nuclear translocation, increasing ICAM-1 and VCAM-1 expression [50]. *Fusobacterium nucleatum*, another key periodontal pathogen, indicates distinct mechanisms of endothelial disruption. FadA adhesin specifically interacts with vascular endothelial–cadherin, while systemic dissemination of *P. gingivalis* occurs through multiple mechanisms [51]. This precise proteolytic activity directly impacts endothelial barrier function. In addition, oxidative stress driven by NOX4 upregulation via TLR2/MyD88 signaling triggers increased superoxide production, resulting in reduced nitric oxide (NO) bioavailability and enhanced lipid peroxidation [18]. This oxidative milieu further impairs endothelial function by inducing direct cellular damage and disrupting vascular homeostasis. The systemic dissemination of *P. gingivalis* occurs through multiple mechanisms that extend far beyond simple bacteriemia.

Direct vascular damage has been comprehensively demonstrated in human umbilical vein endothelial cells (HUVECs). *P. gingivalis* exposure induces endothelial–mesenchymal transformation, with cells losing endothelial markers (CD31, VE-cadherin) while gaining mesenchymal characteristics (α-SMA, vimentin) [52].

Regarding nitric oxide dynamics, periodontitis patients exhibit a striking paradox that directly addresses the reviewer’s concern about NO’s dual nature. Multiple studies confirm significantly elevated iNOS expression in diseased gingival tissues (*p* < 0.001), particularly in macrophages and polymorphonuclear cells [53]. This increased production manifests as elevated nitrite/nitrate levels in gingival crevicular fluid (1.08 ± 0.59 nmol in periodontitis vs 0.83 ± 0.31 nmol in controls) [54].

However, NO metabolites nitrite (NO_2_^−^•) and nitrate (NO_3_^−^) serve as circulating reservoirs and biomarkers in the vasculature [10]

The paradox emerges because bioavailable NO decreases through rapid conversion to peroxynitrite. When iNOS-derived NO reacts with neutrophil-generated superoxide, it forms peroxynitrite at rates exceeding NO’s beneficial signaling. This toxic product nitrosylates antioxidant enzymes, creating a positive feedback loop of oxidative damage [9].

A novel mechanism involves impaired oral nitrate reduction capacity. Recent studies found that periodontitis patients cannot effectively reduce dietary nitrate to nitrite, limiting NO generation from this alternative pathway [55]. Nitrate-reducing bacteria decreased significantly across five countries (r = −0.523 correlation with periodontal pathogens), but recovered after treatment. This discovery links oral dysbiosis directly to systemic NO metabolism dysfunction.

The presence of *P. gingivalis* in atherosclerotic tissues (previously discussed in Section 2) provides compelling evidence for systemic effects. The arterial location-specific detection rates support the systemic dissemination pattern of these pathogens [41].

Bacteraemia frequency depends on oral activities. Professional periodontal procedures generate the highest rates at 46% positive cultures, while daily tooth brushing produces bacteraemia in 10.8–23% of instances [18]. These episodes typically last under 15 min but occur repeatedly, potentially creating three hours of cumulative daily bacteraemia in periodontitis patients. Modern qPCR detection proves significantly more sensitive than traditional culture methods, explaining the historical underestimation of bacteraemia frequency [20].

Importantly, viable *P. gingivalis* bacteria, not just DNA, colonize atherosclerotic tissues. Recent studies demonstrated the presence of living bacteria within vascular tissues using advanced detection techniques [12]. *P. gingivalis* infection correlates with atherosclerotic progression and plaque characteristics [56].

Comprehensive clinical investigations demonstrate variable changes in systemic inflammatory and metabolic markers in periodontitis patients [57]. Surprisingly, these baseline levels did not change significantly 90 days post-treatment, but functional nitrate reduction capacity recovered completely.

Gender-specific differences emerge in larger cohorts. Studies of 89 severe periodontitis patients versus 56 controls found paradoxically lower serum NO metabolites in periodontitis, particularly in males [24]. These levels correlated negatively with C-reactive protein but positively with HDL cholesterol, suggesting complex interactions with systemic inflammation and lipid metabolism.

Endothelial function measurements provide clinically relevant outcomes. A randomized controlled trial demonstrated that periodontal treatment improved endothelial function, with sustained improvements emerging at 60 and 180 days compared to controls (*p* < 0.001) [58]. While acute inflammation initially decreased FMD at 24 h, sustained improvements emerged: 0.9% at 60 days and 2.0% at 180 days compared to controls (*p* < 0.001). These improvements correlated directly with periodontal healing (r = 0.29, *p* = 0.003).

The convergence of these molecular pathways underscores the sophisticated strategies employed by *Porphyromonas gingivalis* to manipulate host cell function. The bacterium’s capacity to simultaneously interfere with multiple cellular processes—including surface receptor activation, intracellular signaling cascades, epithelial barrier integrity, and oxidative stress pathways—positions it as a major contributor to cardiovascular pathology [30]. This orchestrated disruption of endothelial function constitutes a central mechanism linking periodontal disease to CVDs.

## 4. NLRP3 Inflammasome Activation in the Periodontitis–CVDs Link

**Figure 1:**
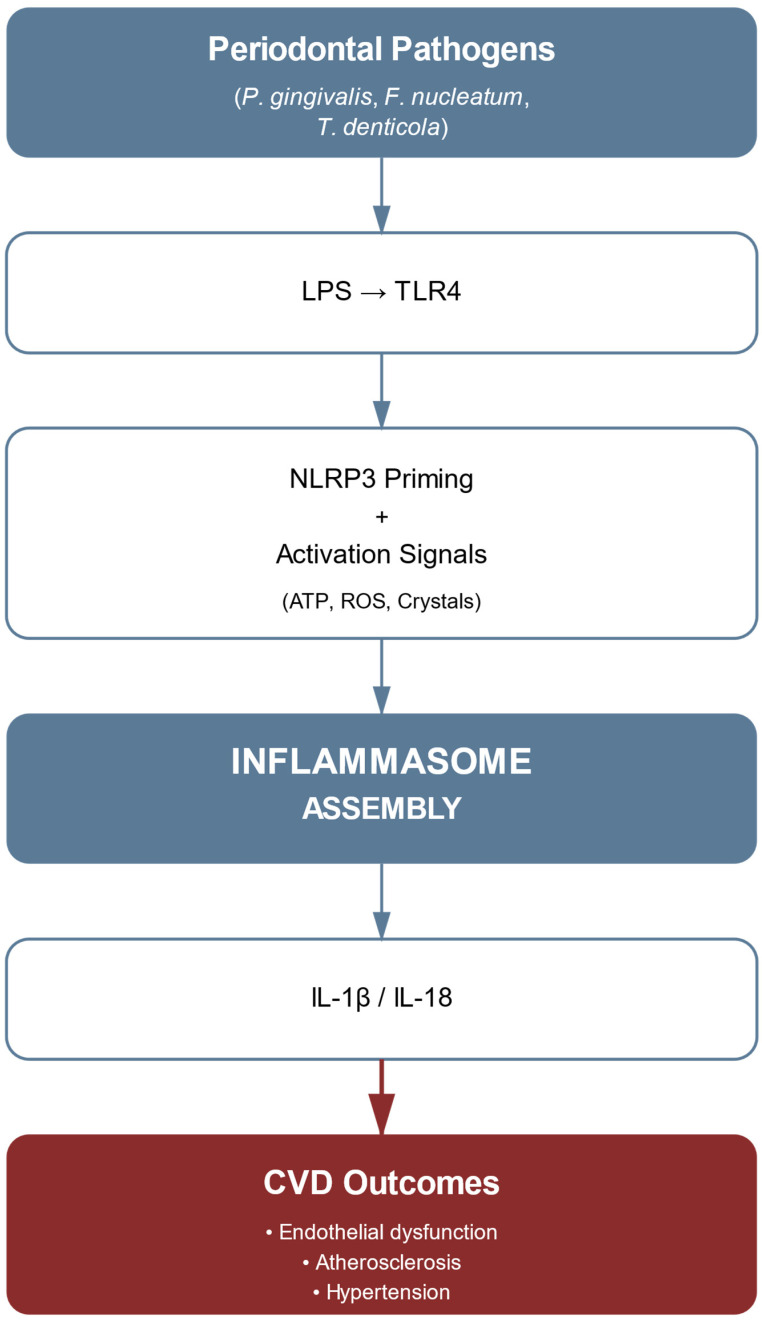
NLRP3 inflammasome pathway linking periodontal pathogens to cardiovascular disease. The cascade shows bacterial recognition through TLR4, two-signal inflammasome activation, cytokine production, and resulting cardiovascular outcomes.

NLRP3 inflammasome emerges as a fundamental molecular nexus in the pathophysiological bridge between periodontal and cardiovascular pathologies. This architecturally sophisticated multiprotein complex—comprising the NLRP3 sensor protein, ASC adaptor molecule, and pro-caspase-1—functions as an intricate intracellular surveillance system for both endogenous and exogenous danger-associated molecular patterns. The following schematic (Figure 1) illustrates the complete NLRP3 inflammasome cascade, from initial bacterial recognition through TLR4 to the ultimate production of mature inflammatory cytokines and their cardiovascular consequences.

As demonstrated in Figure 1, the NLRP3 pathway requires dual signals for activation, highlighting multiple potential therapeutic intervention points. The molecular choreography of NLRP3 activation exhibits remarkable precision through a binary signaling paradigm.

The initial phase is initiated by the interaction of *P. gingivalis* LPS with the TLR4–MD2–CD14 receptor complex at the cellular interface. This interaction orchestrates a sophisticated intracellular signaling cascade involving the adaptor proteins MyD88 and TRIF, ultimately leading to the sequential activation of IRAK1, IRAK4, and TBK1 kinases. These events culminate in the nuclear translocation of NF-κB and the subsequent transcriptional upregulation of both NLRP3 and pro-IL-1β genes [41].

The secondary signal required for full inflammasome activation displays notable molecular versatility, enabling responsiveness to a variety of pathological stimuli within periodontal and cardiovascular microenvironments. Cholesterol crystals, which are abundant in atherosclerotic plaques, represent one major activation stimulus, triggering the inflammasome via lysosomal membrane destabilization and the cytosolic release of cathepsin B. Simultaneously, extracellular ATP released during cell injury provides another activation pathway, stimulating NLRP3 through P2X7 receptor-mediated potassium efflux. This molecular complexity is further enriched by the presence of ROS, derived from both microbial metabolism and host inflammatory responses, which facilitate NLRP3 activation through thioredoxin-interacting protein (TXNIP) dissociation. The convergence of these stimuli forms a tightly regulated molecular network that integrates periodontal and cardiovascular pathophysiology [40]. At the structural level, NLRP3 activation induces conformational changes that expose its pyrin domain (PYD), enabling homotypic PYD–PYD interactions with the adaptor protein ASC. This is followed by CARD–CARD domain interactions that recruit pro-caspase-1, leading to its autocatalytic activation and subsequent cleavage of pro-IL-1β and pro-IL-18 into their mature, bioactive forms [53].

These activated cytokines initiate a multifaceted inflammatory response that profoundly alters vascular homeostasis. IL-1β, a central effector within this axis, mediates a cascade of pleiotropic effects: it promotes endothelial activation via upregulation of adhesion molecules such as VCAM-1 and ICAM-1, thereby facilitating leukocyte adhesion and transmigration [50]. Concurrently, IL-1β drives vascular smooth muscle cell proliferation through intricate intracellular signaling pathways, while also enhancing matrix metalloproteinase expression, thus contributing to extracellular matrix remodeling. The integration of these effects promotes vascular wall destabilization and atherosclerotic plaque progression.

In parallel, IL-18 exerts distinct immunomodulatory functions, primarily by inducing interferon-γ production in T lymphocytes and natural killer cells. This action perpetuates a feedforward loop of systemic inflammation, thereby amplifying cardiovascular risk [40].

Recent molecular investigations have revealed that NLRP3 inflammasome activation in periodontal disease extends well beyond local tissue responses. When activated within endothelial and vascular smooth muscle cells, this molecular platform promotes endothelial dysfunction and accelerates atherogenesis. Sustained inflammasome activity fosters a persistent pro-inflammatory milieu, effectively bridging the pathological continuum between oral and CVDs [41].

Recognition of the NLRP3 inflammasome as a central molecular hub in this pathophysiological axis has profound therapeutic implications. Given its upstream position within the inflammatory cascade, NLRP3 represents an appealing target for selective pharmacological intervention. Inhibition of this platform could disrupt the bidirectional inflammatory communication between the periodontal and cardiovascular systems, offering dual clinical benefits [30].

Furthermore, NLRP3 activation status has emerged as a potential biomarker for cardiovascular risk stratification in patients with periodontitis. Its systemic relevance highlights the broader implications of localized oral inflammation and reinforces the need for integrated management approaches [31].

Contemporary therapeutic strategies reflect an unprecedented level of molecular specificity in targeting the NLRP3 cascade, marking a paradigm shift in the treatment of periodontal–cardiovascular comorbidity. Selective NLRP3 inhibitors precisely disrupt key protein–protein interactions within the inflammasome complex. In parallel, upstream modulators target signal initiation pathways, while downstream interventions focus on cytokine processing and release. This multi-tiered therapeutic architecture allows for coordinated interference at several regulatory checkpoints within the inflammasome pathway [31].

The integration of these targeted approaches offers a refined strategy capable of addressing both local periodontal inflammation and its systemic cardiovascular consequences. By modulating critical molecular nodes, these interventions surpass traditional anti-inflammatory therapies in both specificity and efficacy. Emerging evidence supports their potential as transformative tools in the clinical management of patients with concurrent periodontal and CVDs [40].

## 5. Oxidative Stress Mechanisms in Periodontitis-Associated Cardiovascular Disease: From ROS Generation to Vascular Dysfunction

Oxidative stress significantly mediates the relationship between periodontitis and CVDs. Generated superoxide undergoes several transformations critical to pathogenesis, while nitric oxide signaling experiences significant perturbation. NOX2, a primary member of the NADPH oxidase family, initiates the oxidative cascade in periodontal inflammation. Expressed predominantly in phagocytes, NOX2 generates superoxide (O_2_•^−^) through electron transfer from NADPH to molecular oxygen, leading to significant increases in ROS production in inflamed sites [47,48]. This dramatic elevation in ROS production initiates several transformations critical to pathogenesis: Superoxide dismutase (SOD) converts O_2_•^−^ to hydrogen peroxide (H_2_O_2_), which indicates membrane permeability and participates in the Fenton reaction, producing highly reactive hydroxyl radicals (•OH). Additionally, myeloperoxidase (MPO) catalyzes the formation of hypochlorous acid (HOCl) from H_2_O_2_, contributing to tissue damage despite its antimicrobial properties [33]. The nitric oxide signaling cascade undergoes significant perturbation during periodontal inflammation through precisely characterized molecular mechanisms.

While endothelial nitric oxide synthase (eNOS) constitutively produces NO for the maintenance of vascular homeostasis, the pathological accumulation of superoxide initiates a deleterious sequence: the rapid reaction between NO and superoxide generates peroxynitrite (ONOO−) that accumulates in affected vascular tissues [9,10]. These concentrations, representing substantial increases over physiological levels, significantly reduce NO bioavailability [10,59]. The uncoupled enzyme subsequently generates superoxide rather than NO, establishing a feed-forward cycle of oxidative stress. The impact on lipid metabolism manifests through specific oxidative modifications: periodontal pathogen-enhanced low-density lipoprotein (LDL) oxidation proceeds through sequential molecular events, beginning with hydrogen abstraction from lipids, followed by lipid peroxyl radical formation through molecular oxygen interaction. These reactive intermediates initiate chain reactions producing reactive aldehydes, particularly malondialdehyde (MDA) and 4-hydroxynonenal (4-HNE), which form specific adducts with apolipoprotein B-100. The modification of LDL particles results in documented increases in MDA-LDL levels and significant elevation in 4-HNE-modified proteins in plasma [50]. These modified lipoproteins demonstrate enhanced uptake by macrophages, substantially increasing foam cell formation compared to native LDL [60]. The endogenous antioxidant defence system exhibits systematic dysregulation in periodontitis patients with cardiovascular complications, characterized by quantifiable perturbations in multiple molecular pathways. Contemporary analyses have documented precise alterations in redox homeostasis: plasma superoxide dismutase activity and vitamin C levels show reductions compared to healthy controls [9,11]. These reductions in antioxidant defences contribute directly to the molecular pathogenesis linking periodontal and CVDs. These quantifiable reductions in antioxidant defenses contribute directly to the molecular pathogenesis linking periodontal and CVDs, showing remarkable complexity in oxidative stress mechanisms, where reactive ROS and RNS orchestrate a sophisticated cascade of pathophysiological events.

**Figure 2:**
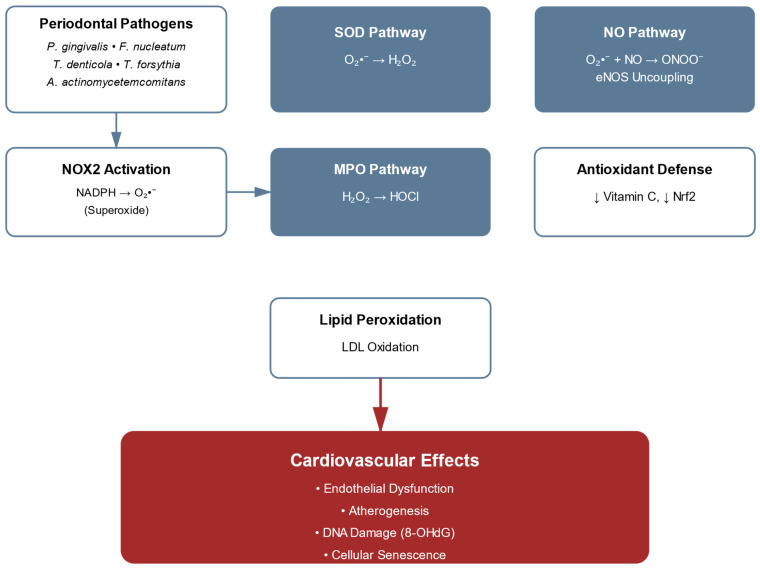
Oxidative stress pathways in periodontitis-associated cardiovascular disease. The diagram illustrates the complex oxidative stress mechanisms linking periodontal pathogens to cardiovascular pathology. Periodontal pathogens (*P. gingivalis*, *F. nucleatum*, *T. denticola*, *T. forsythia*, *A. actinomycetemcomitans*) initiate NOX2 activation, leading to superoxide (O_2_•^−^) production via NADPH. This branches into three major pathways: (1) SOD pathway converting O_2_•^−^ to H_2_O_2_, (2) MPO pathway producing HOCl from H_2_O_2_, and (3) NO pathway showing the interaction between O_2_•^−^ and NO to form ONOO−, resulting in eNOS uncoupling. The diagram also depicts the antioxidant defense mechanisms (downregulation of Vitamin C and Nrf2) and lipid peroxidation processes (LDL oxidation). These pathways converge to produce cardiovascular effects, including endothelial dysfunction, atherosclerosis, DNA damage (8-OHdG), and cellular senescence. Note: ↓ indicates decrease compared to healthy controls or baseline values.

This intricate molecular network initiates with specific triggers, notably periodontal pathogens and inflammatory mediators, which activate precise enzymatic systems generating reactive species. The subsequent molecular events encompass carefully regulated oxidative modifications of cellular components, ultimately culminating in defined vascular consequences. Figure 2 provides a comprehensive overview of the oxidative stress mechanisms, illustrating how periodontal pathogens initiate NOX2 activation and the subsequent branching pathways that lead to endothelial dysfunction.

This oxidative cascade exhibits remarkable specificity in both its initiation and propagation, with each step representing a potential therapeutic target in the management of periodontitis-associated cardiovascular complications.

The pathways depicted in Figure 2 reveal three critical nodes where oxidative damage occurs: the SOD pathway, the MPO pathway, and the NO/peroxynitrite interaction, each representing potential therapeutic targets. The Nrf2 signaling pathway, central to antioxidant response regulation, manifests specific molecular aberrations. While oxidative stress normally triggers Nrf2 release from its cytoplasmic anchor Keap1 through modification of specific cysteine residues, chronic periodontal inflammation impairs this protective mechanism [9]. Chronic inflammatory states have been shown to downregulate Nrf2 target gene expression, with significant effects on HO-1 (heme oxygenase-1) and NQO1 (NAD(P)H quinone dehydrogenase 1) [9,15]. This reduction correlates with elevated oxidative stress markers. DNA damage emerges as a consequence of these oxidative perturbations, evidenced by increased levels of 8-hydroxy-2′-deoxyguanosine (8-OHdG) in patients with severe periodontitis and concomitant CVDs [10,11]. These changes are associated with activation of cellular senescence pathways [15,61]. The molecular alterations in oxidative pathways manifest specific impacts on vascular function. In periodontal tissues, myeloperoxidase activity demonstrates a significant increase, with corresponding elevation of HOCl-modified proteins and oxidized LDL [10,60]. The endothelial dysfunction cascade exhibits measurable perturbations: peroxynitrite formation reduces BH4 bioavailability, resulting in eNOS uncoupling and a shift from NO production to superoxide generation [9,10,59]. Recent analyses have identified oxidative modifications in vascular tissues, with periodontal inflammation increasing protein carbonyl content in affected vessels [9,60]. These modifications correlate with increased expression of inflammatory adhesion molecules, including VCAM-1 and ICAM-1 [50,62]. Table 1 systematically documents these alterations, providing reference ranges and clinical significance for key biomarkers in periodontitis-associated CVDs, establishing quantitative parameters for therapeutic monitoring and intervention strategies.

The oxidative burden manifests in quantifiable DNA modifications, as evidenced by elevated 8-hydroxy-2′-deoxyguanosine (8-OHdG) levels in individuals with severe periodontitis and concurrent cardiovascular pathology [10,11]. This genomic instability is accompanied by alterations in cellular senescence pathways [61].

The molecular interface between periodontal and cardiovascular pathologies is characterized by alterations in oxidative enzymology. In affected tissues, myeloperoxidase exhibits enhanced catalytic activity with concomitant elevation of HOCl-modified proteins [10,60]. Endothelial dysfunction manifests through molecular perturbations, including peroxynitrite-mediated oxidation that reduces tetrahydrobiopterin (BH4) bioavailability, contributing to eNOS uncoupling and increased superoxide generation [10,59].

Contemporary analyses have identified increased protein carbonyl content in the vascular milieu associated with periodontal inflammation [9,60], with preferential accumulation in endothelial and vascular smooth muscle compartments. These post-translational modifications demonstrate direct correlation with enhanced expression of inflammatory adhesion molecules.

## 6. Systemic Inflammatory Response to Periodontal Pathogens

The critical pathophysiological nexus between periodontal pathogens and CVDs is mediated by intricate inflammatory cascades that extend far beyond the confines of the oral cavity. Contemporary molecular research has delineated specific signal transduction pathways through which key periodontal pathogens—*Porphyromonas gingivalis*, *Aggregatibacter actinomycetemcomitans*, *Fusobacterium nucleatum*, and *Treponema denticola*—orchestrate systemic inflammatory responses [28,33,34].

These microorganisms exploit highly evolved molecular delivery mechanisms, notably outer membrane vesicles (OMVs), which function as efficient vectors for the dissemination of virulence factors. Ranging in size from 20 to 250 nm, these nanoscale vesicles possess the capacity for hematogenous dissemination and interact with pattern recognition receptors (PRRs) across the vasculature, thereby initiating inflammatory cascades in anatomically distant tissues [37]. 

The resultant inflammatory response is reflected in quantifiable alterations in systemic cytokine profiles. Patients with severe periodontitis exhibit significantly elevated levels of pro-inflammatory mediators—namely IL-1β, TNF-α, and IL-6—in both serum and saliva. These elevated cytokines exert direct effects on vascular homeostasis and atherogenesis through a variety of well-characterized molecular mechanisms [39].

Disruption of lipid homeostasis has emerged as a critical mechanistic intersection between periodontitis and cardiovascular pathology. *P. gingivalis*-derived LPS induces specific post-translational modifications in LDL particles, enhancing their atherogenic properties. These modified LDL particles exhibit increased recognition and uptake by macrophage scavenger receptors, thereby accelerating foam cell formation and the progression of atherosclerotic lesions. In parallel, high-density lipoprotein (HDL) undergoes functional impairment, manifesting as diminished anti-inflammatory capacity and reduced cholesterol efflux potential [50].

The complement cascade, a key component of innate immunity, is significantly modulated through pathogen-driven mechanisms. Of particular importance is the activity of *P. gingivalis* gingipains, which exhibit remarkable substrate specificity in cleaving complement component C5. This results in the generation of biologically active C5a through a non-canonical pathway, representing a fundamental deviation from classical complement activation. This aberrant activation fosters a pro-inflammatory microenvironment with systemic implications, particularly for vascular integrity and function [47].

Recent studies have also elucidated the role of neutrophil extracellular traps (NETs) as a critical mechanistic link between periodontal and cardiovascular disease. In periodontitis, *P. gingivalis* triggers NETosis through gingipain-mediated cleavage of PAR-2 receptors, initiating calcium mobilization and NADPH oxidase activation [31]. This process requires PAD4 (peptidylarginine deiminase 4) for histone citrullination, which converts positively charged arginine residues to neutral citrulline, promoting chromatin decondensation and NET release [63].

Quantitative evidence demonstrates significant NET elevation in periodontitis patients. Cell-free DNA levels are 5-fold higher in saliva (582 ± 1023 ng/mL vs. 100 ± 259 ng/mL, *p* < 0.001), while citrullinated histone H3 is detected in 60% of periodontal tissues [64]. Building on these local markers, MPO-DNA complexes representing intact NET structures show elevated levels in circulation [65].

These NETs serve multiple pathogenic roles in cardiovascular pathology. They provide a three-dimensional scaffold for platelet adhesion through histone–platelet interactions, activate the contact pathway of coagulation as the DNA backbone triggers Factor XII, and promote tissue factor expression on monocytes through inflammatory mediator release [30]. NET-associated proteases, including neutrophil elastase, degrade natural anticoagulants such as tissue factor pathway inhibitor and thrombomodulin, creating a persistent prothrombotic surface [66].

Clinical studies demonstrate that circulating NET markers correlate with cardiovascular risk in periodontitis patients. Cell-free DNA and citrullinated histone H3 levels associate with both periodontal severity and cardiovascular events, with the PAROKRANK follow-up study showing adjusted hazard ratios of 1.26 for future cardiovascular events in periodontitis patients [67]. These findings position NETs as both biomarkers and therapeutic targets, with emerging interventions including PAD4 inhibitors like GSK484, DNase treatment, and peptide inhibitors such as neonatal NET-inhibitory factor showing promise in preclinical models [68,69].

Endothelial homeostasis is notably disrupted through multiple molecular pathways, including the enhanced biosynthesis of ADMA, as detailed in Section 7. Periodontal inflammation enhances the biosynthesis of ADMA, a potent endogenous inhibitor of endothelial eNOS. This results in reduced NO production and the establishment of a self-perpetuating cycle of endothelial dysfunction, ultimately compromising vascular competence [42].

The cumulative effects of these inflammatory mechanisms extend to profound phenotypic modulation of vascular smooth muscle cells (VSMCs). Periodontal pathogens induce a transition from a contractile to a synthetic phenotype in VSMCs, a process mediated by sophisticated epigenetic modifications and alterations in transcriptional programs. This phenotypic plasticity promotes vascular remodelling and contributes significantly to the advancement of atherosclerosis [55].

These molecular insights have led to the identification of precise therapeutic targets amenable to pharmacological intervention. Emerging strategies have focused on the selective modulation of inflammatory pathways. For instance, targeted inhibition of gingipain–C5 interactions has demonstrated promising anti-inflammatory effects, including reduced expression of vascular adhesion molecules and decreased monocyte recruitment in experimental models [33].

NET formation has likewise emerged as a compelling therapeutic target. Strategies aimed at modulating NETosis—while preserving essential neutrophil functions—have shown efficacy in attenuating both periodontal inflammation and its cardiovascular sequelae. Engineered peptide inhibitors of NET formation have been particularly effective in preclinical models, significantly reducing both local periodontal tissue damage and systemic markers of vascular inflammation [31].

Regulation of ADMA metabolism represents another critical therapeutic opportunity. Recent studies have demonstrated that interventions targeting enzymes responsible for ADMA catabolism can improve endothelial function. Strategies aimed at enhancing ADMA degradation or inhibiting its synthesis have shown promise in restoring endothelial homeostasis and reducing cardiovascular risk in patients with periodontitis [42].

In addition, the modulation of lipid metabolism has gained attention as a novel therapeutic strategy. Efforts to enhance HDL functionality and prevent pathogen-mediated LDL modification have demonstrated considerable potential in mitigating atherosclerotic progression. These strategies include the development of specific inhibitors targeting bacterial enzymes responsible for lipoprotein modification, as well as compounds designed to augment the anti-inflammatory properties of HDL [50].

Table 2 offers a detailed overview of the key molecular mediators and their altered expression in periodontitis, along with their respective impacts on cardiovascular health.

Emerging evidence suggests profound therapeutic potential in targeting epigenetic modifications induced by periodontal pathogens. Specifically engineered epigenetic modulators have demonstrated efficacy in preventing pathogen-induced phenotypic transformation of vascular cells while concurrently attenuating inflammatory gene expression [55]. This epigenetic intervention paradigm represents a sophisticated approach to disrupting the molecular mechanisms linking periodontal and cardiovascular pathologies.

The therapeutic implications of these molecular insights extend beyond singular interventions, suggesting the potential efficacy of coordinated therapeutic approaches targeting multiple pathogenic mechanisms simultaneously. Such integrated strategies, addressing both inflammatory cascades and their downstream molecular effectors, may provide more comprehensive therapeutic outcomes in managing the cardiovascular sequelae of periodontal disease.

## 7. The Role of ADMA in Periodontal–Cardiovascular Pathology

Asymmetric dimethylarginine (ADMA), an endogenous methylated derivative of L-arginine, functions as a critical molecular mediator linking periodontal disease to cardiovascular dysfunction through endothelial modulation. ADMA originates from the proteolysis of proteins containing methylated arginine residues, with methylation catalyzed by protein arginine methyltransferases (PRMTs) types I and II [71,72,73]. Structurally, ADMA differs from L-arginine only by two methyl groups on the terminal guanidino nitrogen, yet this modification enables ADMA to act as a competitive inhibitor of nitric oxide synthase (NOS) isoforms [70,74]. Systematic clinical investigations demonstrate that patients with periodontal disease exhibit significantly elevated circulating ADMA concentrations compared to matched controls, establishing a quantitative basis for this association [58,73,75].

The molecular mechanisms underlying ADMA elevation in periodontitis follow a tightly orchestrated sequence. LPS derived from Porphyromonas gingivalis activates NF-κB signaling, resulting in the upregulation of inflammatory pathways [76]. This process is accompanied by cytokine-mediated suppression of dimethylarginine dimethylaminohydrolase (DDAH), the enzyme primarily responsible for ADMA catabolism. Concurrently, elevated reactive oxygen species (ROS) in periodontal tissues can compromise DDAH enzymatic activity, perpetuating a self-reinforcing cycle of oxidative stress and ADMA accumulation [59,77].

ADMA exerts its pathogenic influence on the endothelium through well-documented mechanisms: high-affinity competitive inhibition of eNOS, leading to a significant reduction in nitric oxide (NO) bioavailability [73,78]. This mechanism assumes particular pathophysiological significance when ADMA levels exceed physiological concentrations, precipitating eNOS uncoupling and superoxide generation. The dose-dependent association between periodontal disease indices and ADMA has been documented in untreated hypertensive patients [79,80]. Clinical trials report concomitant improvements in vascular function among patients with coexisting periodontal and CVDs [51]. Antioxidant compounds that prevent DDAH inactivation have demonstrated efficacy in preserving enzymatic activity and mitigating ADMA accumulation [81].

**Figure 3:**
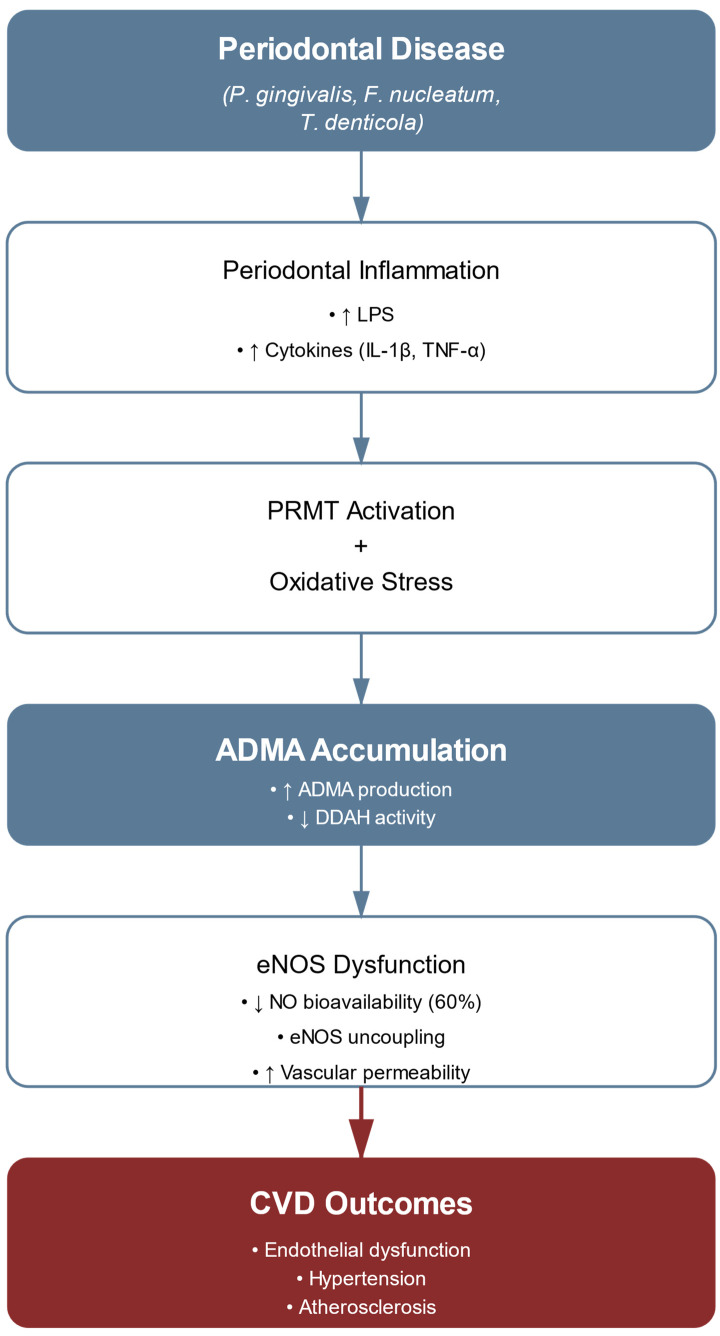
The role of ADMA in periodontal–cardiovascular pathology. Schematic representation of ADMA’s pathophysiological role in linking periodontal disease to cardiovascular complications. The pathway initiates with periodontal disease and subsequent inflammation, branching into two primary mechanisms: *P. gingivalis* LPS-induced protein arginine methyltransferases (PRMT) upregulation and oxidative stress-mediated ROS production. These pathways converge to influence ADMA levels through increased production and decreased clearance (via DDAH inhibition). The resulting ADMA accumulation leads to three major molecular effects: eNOS inhibition (reducing NO production), eNOS uncoupling (causing oxidative stress), and Rho/ROCK activation (increasing vascular permeability). These mechanisms ultimately culminate in cardiovascular pathology characterized by endothelial dysfunction, hypertension, and atherosclerosis. Arrows indicate directional relationships, with ↑ representing increase and ↓ representing decrease in activity or production.

Advanced therapeutic strategies now target multiple molecular nodes within the ADMA pathway. These include the use of selective small-molecule PRMT inhibitors and nanoparticle-based delivery systems that enhance DDAH stability and activity. Targeted interventions have also focused on reversing ADMA-mediated phenotypic modulation of vascular smooth muscle cells (VSMCs). Figure 3 presents a schematic representation of ADMA’s central role in linking periodontal inflammation to cardiovascular dysfunction, highlighting the dual mechanisms of increased ADMA production and decreased clearance. The rapidly evolving field of epigenetic therapeutics continues to yield increasingly sophisticated molecular tools for the precise modulation of pathological gene expression programs.

## 8. Epigenetic Modifications in Periodontal–Cardiovascular Pathology

The interplay between periodontal and cardiovascular diseases is further supported by sophisticated epigenetic modifications that extend beyond canonical inflammatory pathways. These heritable yet reversible alterations in gene expression, occurring independently of DNA sequence changes, offer critical insights into the long-term cardiovascular sequelae of periodontal inflammation [15,82].

Among these, the ANRIL/CDKN2B-AS1 locus represents a well-established genetic risk factor implicated in both periodontitis and cardiovascular diseases [83,84,85]. The study by Schaefer et al. identified genetic polymorphisms at this locus that confer shared susceptibility for coronary heart disease and periodontitis, establishing a genetic basis for the observed association between these conditions [86].

Histone modifications play an essential role. *P. gingivalis* induces specific epigenetic modifications in vascular endothelial cells, promoting sustained transcription of pro-inflammatory genes and contributing to chronic vascular inflammation in periodontitis patients [82,87]. DNA methylation changes have been documented to underlie the long-term association between periodontitis and atherosclerotic cardiovascular disease [88].

Long non-coding RNAs (lncRNAs) have emerged as central regulators of these epigenetic processes. The CDKN2B-AS1 lncRNA has been shown to regulate collagen expression, potentially contributing to vascular remodeling [85]. *P. gingivalis* triggers durable epigenetic reprogramming in monocytic populations, establishing a persistent, hyper-responsive cellular phenotype—a phenomenon emblematic of trained immunity in the context of periodontal–cardiovascular pathophysiology [82]. Recent reviews have explored targeting epigenetic plasticity to reduce periodontitis-related inflammation, including the potential of natural products such as CBD, metformin, and polyphenolic compounds as epigenetic modulators [89]. These interventions offer promising non-invasive therapeutic adjuncts for managing the periodontal–cardiovascular disease continuum. The integration of epigenetic biomarkers into clinical diagnostics presents a transformative opportunity for personalized medicine. DNA methylation profiles and other epigenetic signatures have demonstrated substantial prognostic value in understanding the molecular mechanisms linking periodontitis to cardiovascular disease, thereby facilitating early intervention and tailored therapeutic strategies [88].

## 9. Molecular Mechanisms of Periodontal Pathogen-Induced Vascular Smooth Muscle Cell Dysfunction: From Phenotypic Switching to Therapeutic Targets

Vascular smooth muscle cells (VSMCs) are central regulators of vascular homeostasis and key contributors to the pathogenesis of atherosclerosis. Recent molecular studies have delineated complex mechanistic pathways through which periodontal pathogens disrupt VSMC function, thereby contributing to cardiovascular pathology.

The phenotypic plasticity of VSMCs—particularly their transition from a contractile to a synthetic phenotype—constitutes a critical molecular event in the progression of atherosclerosis. Porphyromonas gingivalis-derived outer membrane vesicles (OMVs) initiate this phenotypic shift via activation of the ERK1/2-RUNX2 signaling cascade, resulting in elevated expression of synthetic markers such as osteopontin and osteogenic markers, alongside enhanced VSMC migratory and proliferative capacities [90].

Metabolic reprogramming has emerged as a central determinant of VSMC dysfunction. Periodontal pathogens promote a shift toward glycolytic metabolism through activation of the hypoxia-inducible factor 1-alpha (HIF-1α) pathway, thereby increasing glucose uptake and lactate production. This metabolic adaptation enhances VSMC proliferation and migration within the inflammatory vascular milieu [91].

Epigenetic regulation plays a fundamental role in sustaining VSMC dysfunction. Chronic exposure to *P. gingivalis* LPS induces stable transcriptional reprogramming via specific histone modifications, most notably H3K4me3 enrichment at pro-inflammatory gene loci [92].

The molecular basis of vascular calcification emerges as a significant consequence of periodontal pathogen–VSMC interaction. A. actinomycetemcomitans-derived cytolethal distending toxin promotes mineralization through NLRP3 inflammasome activation, precipitating increased IL-1β production and osteogenic gene expression [93].

MicroRNA-mediated regulation, particularly through miR-21, functions as a critical molecular arbiter of VSMC dysfunction. While miR-21 is known to modulate inflammatory responses and its relationship with PTEN/PI3K/AKT signaling is established in vascular cells, direct evidence linking *P. gingivalis* infection to this pathway in VSMCs requires further validation [94].

Autophagic dysfunction represents a crucial mechanism through which periodontal pathogens modulate VSMC function. Chronic pathogen exposure has been shown to affect autophagy pathways, though the specific effects on ATG5 and BECN1 in VSMCs remain to be fully characterized [95].

Pathogen-derived extracellular vesicles (EVs) demonstrate sophisticated modulation of VSMC phenotype through precise molecular mechanisms. These nanoscale structures (30–150 nm diameter) deliver specific bacterial small RNAs that can modulate host gene expression, potentially influencing VSMC phenotypic plasticity [96].

**Figure 4:**
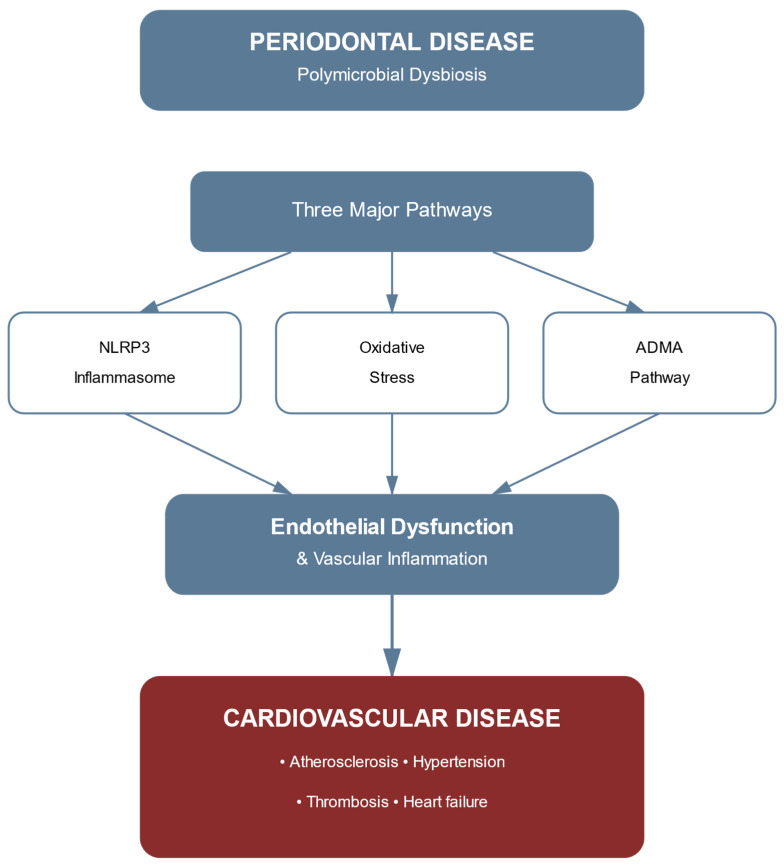
Integrated pathways: periodontitis to CVDs. Schematic representation showing how periodontal disease (polymicrobial dysbiosis) leads to cardiovascular disease through three major molecular pathways: NLRP3 inflammasome, oxidative stress, and ADMA pathway. These mechanisms converge to cause endothelial dysfunction and vascular inflammation, ultimately resulting in cardiovascular pathology, including atherosclerosis, hypertension, thrombosis, and heart failure.

Figure 4 provides a comprehensive summary of the integrated molecular pathways linking periodontitis to cardiovascular disease through the three major mechanisms discussed throughout this review: NLRP3 inflammasome activation, oxidative stress pathways, and ADMA-mediated endothelial dysfunction. These pathways converge to promote endothelial dysfunction and vascular inflammation, ultimately culminating in cardiovascular disease manifestations.

Proteomic analyses have identified coordinated modulation of over 200 proteins in response to periodontal pathogen exposure, with significant enrichment in pathways related to cellular migration, metabolism, and inflammation [30].

These mechanistic insights inform the development of targeted therapeutic strategies, including the following:

- Antagonism of microRNA pathways (e.g., miR-21 inhibition);
- Restoration of autophagic activity;
- Modulation of EV-mediated signaling;
- Epigenetic reprogramming to reverse phenotypic plasticity [38].

The elucidation of these molecular mechanisms provides a robust conceptual framework for the development of precision-targeted therapies aimed at mitigating periodontitis-associated vascular complications [31].

## 10. Therapeutic Implications and Clinical Translation

The molecular pathways elucidated throughout this review have informed the development of targeted therapeutic strategies for managing the periodontal–cardiovascular disease continuum. These interventions range from optimization of conventional periodontal treatments to sophisticated molecular approaches targeting specific pathogenic mechanisms (Table 3). The translation of mechanistic insights into clinical applications represents a critical step toward integrated patient management.

The current evidence suggests that multi-modal approaches combining mechanical periodontal therapy with molecular interventions may offer superior outcomes compared to monotherapy. Future therapeutic development will likely focus on personalized treatment selection based on individual molecular profiles and disease phenotypes.

## 11. Research Gaps and Future Directions

Despite significant advances in elucidating the molecular mechanisms linking periodontal disease to cardiovascular pathology, critical gaps remain in translating these discoveries into clinical practice. The most pressing need is for longitudinal studies that track molecular biomarkers prospectively to establish causal relationships between specific inflammatory mediators and cardiovascular events. Current evidence largely relies on cross-sectional associations, limiting our ability to predict disease progression. Future research should prioritize the development of integrated multi-omics approaches combining genomic, proteomic, and metabolomic data to create personalized risk stratification models. Additionally, the optimal therapeutic window for intervention remains undefined—determining whether early molecular changes can serve as actionable targets before clinical manifestations emerge represents a key challenge. The rapid advancement of point-of-care diagnostics and machine learning algorithms offers unprecedented opportunities to identify high-risk patients who would benefit most from targeted interventions. Finally, while individual molecular pathways have been well characterized, understanding their complex interactions within the broader systems biology context will be essential for developing truly effective therapeutic strategies that address the periodontal–cardiovascular disease continuum.

## 12. Conclusions

This comprehensive review consolidates current knowledge on the molecular mechanisms underpinning the association between periodontal disease and cardiovascular pathology. The evidence presented underscores the intricate interplay between oral pathogens—*P. gingivalis* in particular—and vascular dysfunction, mediated through multiple converging pathways including NLRP3 inflammasome activation, oxidative stress induction, and epigenetic reprogramming. Notably, the oral microbiome exerts effects far beyond the confines of the oral cavity, contributing to a systemic pro-inflammatory milieu that facilitates atherogenesis and endothelial impairment. These insights reinforce the critical importance of maintaining oral health as an integral component of CVD prevention and unveil novel molecular targets for therapeutic intervention. Moving forward, research efforts should prioritize the development of integrated, precision-based therapies that concurrently address the periodontal and cardiovascular dimensions of this pathophysiological continuum.

## Figures and Tables

**Table 1:** Biomarkers and their clinical significance in periodontitis–CVDs. Biomarkers and their clinical significance in periodontitis–CVDs. Data compiled from [42] for vitamin C and SOD activity; [46] for peroxynitrite; [57] for ADMA; [42,58] for VCAM-1; [50,62] for CRP; [42,49] for NO bioavailability; [49] for BH4 levels; [57] for flow-mediated dilation. Note: ↑ indicates increase; ↓ indicates decrease compared to healthy controls or baseline values. Fold-changes and percentages represent mean values from published studies.

Biomarker Category	Marker	Normal Range	Pathological Level	Clinical Significance
Oxidative stress	Vitamin C	45–80 μM	↓	Antioxidant depletion
	SOD activity	785–1570 U/g Hb	↓	Impaired ROS neutralization
	Peroxynitrite	<0.1 μM	0.5–1.0 μM	eNOS uncoupling
Inflammation	ADMA	0.4–0.6 μM	>0.8 μM (elevated)	Endothelial dysfunction
	VCAM-1	400–800 ng/mL	↑	Vascular inflammation
	CRP	<3 mg/L	>5 mg/L	Systemic inflammation
Vascular function	NO bioavailability	>85%	↓	Endothelial dysfunction
	BH4 levels	5–15 nM	↓	eNOS cofactor depletion
	Flow-mediated dilation	>10%	<7%	Impaired vascular reactivity

**Table 2:** Key molecular mediators in periodontitis-associated cardiovascular disease. Data compiled from: [39,40,70] for inflammatory cytokines; [47,48,50,61] for oxidative stress markers; [15,17,29] for epigenetic regulators. Note: ↑ indicates increase; Fold changes and percentages represent mean values from published studies.

Clinical Reference Values	Major Cardiovascular Effects	Changes in Periodontitis	Molecule	Mediator Category
				**Inflammatory cytokines**
Normal: <3.5 pg/mL	Endothelial dysfunction, VCAM-1 upregulation	↑ in serum	IL-1β	
Normal: <10 pg/mL	Atherosclerotic plaque formation, VSMC proliferation	↑ in serum	TNF-α	
Normal: <5 pg/mL	Plaque instability, CRP production	↑ in serum	IL-6	
				**Oxidative stress markers**
Reference: Basal activity	Sustained ROS generation, endothelial damage	↑ activity	NOX2	
Normal: <2.0 ng/mL	DNA oxidation marker, cellular damage	↑ in serum	8-OHdG	
Normal: <50 U/L	Foam cell formation, plaque progression	↑ in plasma	MDA-LDL	
				**Epigenetic regulators**
Baseline expression	Inflammatory pathway regulation, NF-κB signaling	↑ expression	miR-146a	
ChIP enrichment ratio	Pro-inflammatory gene activation	↑ at promoters	H3K4me3	
Enzymatic activity units	Altered DNA methylation patterns	↑ activity	DNMT1	

**Table 3:** Current and emerging therapeutic strategies for periodontal–cardiovascular disease management.

Treatment Category	Specific Intervention	Mechanism of Action	Development Stage	Clinical Evidence
Conventional periodontal	Scaling and root planing	Mechanical disruption of biofilm, reduction of bacterial load	Standard of care	35% reduction in CRP, improved FMD
	Antimicrobial therapy	Direct bacterial elimination	Clinical use	Variable efficacy, resistance concerns
Anti-inflammatory	MCC950 (NLRP3 inhibitor)	Selective NLRP3 inflammasome blockade	Phase II trials	Reduced IL-1β, IL-18 in pilot studies
	Canakinumab (IL-1β antibody)	IL-1β neutralization	FDA approved (other indications)	CANTOS trial: 15% CVDs risk reduction
Antioxidant	NOX2 inhibitors (GSK2795039)	Targeted ROS reduction	Preclinical	70% reduction in vascular superoxide
	Mitochondria-targeted antioxidants	Cellular oxidative stress reduction	Phase I	MitoQ shows promise in animal models
Epigenetic modulators	HDAC inhibitors	Reversal of pathogenic gene expression	Phase I/II	Restoration of eNOS expression
	DNMT inhibitors	DNA methylation modification	Preclinical	KLF4 promoter demethylation achieved
Microbiome-based	*S. salivarius* M18 probiotic	Competitive exclusion, bacteriocin production	Commercial availability	40% reduction in pathogen load
	Gingipain inhibitors	Specific virulence factor targeting	Preclinical	COR388 in development
ADMA-targeted	L-citrulline supplementation	Enhanced ADMA clearance	Clinical use	30% ADMA reduction, improved FMD
	DDAH enhancers	Increased ADMA metabolism	Preclinical	Proof-of-concept established
Combination approaches	Periodontal therapy + statins	Synergistic anti-inflammatory effects	Observational studies	Enhanced CVD risk reduction
	Omega-3 + periodontal treatment	Resolution of inflammation	Small RCTs	Improved clinical outcomes

Abbreviations: CRP, C-reactive protein; FMD, flow-mediated dilation; FDA, Food and Drug Administration; CANTOS, Canakinumab Anti-inflammatory Thrombosis Outcomes Study; RCT, randomized controlled trial.

## Data Availability

The authors confirm that the data supporting the findings of this study are available within the article.

## Abbreviations

ADMA: Asymmetric Dimethylarginine; ASC, Apoptosis-associated Speck-like protein containing a CARD; ATP, Adenosine Triphosphate; BET, Bromodomain and Extra-terminal; BH4, Tetrahydrobiopterin; CARD, Caspase Recruitment Domain; CDV, Cardiovascular Disease; CRP, C-Reactive Protein; DDAH, Dimethylarginine Dimethylaminohydrolase; DNMT1, DNA Methyltransferase 1; eNOS, Endothelial Nitric Oxide Synthase; EndMT, Endothelial-to-Mesenchymal Transition; EV, Extracellular Vesicle; H_2_O_2_, Hydrogen Peroxide; HDL, High-Density Lipoprotein; HIF-1α, Hypoxia-Inducible Factor 1-alpha; HO-1, Heme Oxygenase-1; HOCl, Hypochlorous Acid; HSP, Heat Shock Protein; ICAM-1, Intercellular Adhesion Molecule-1; IL, Interleukin; IRAK, Interleukin-1 Receptor-Associated Kinase; LDL, Low-Density Lipoprotein; lncRNA, Long Non-coding RNA; LPS, Lipopolysaccharide; MAPK, Mitogen-Activated Protein Kinase; MDA, Malondialdehyde; MMP, Matrix Metalloproteinase; MPO, Myeloperoxidase; MyD88, Myeloid Differentiation primary response 88; NADPH, Nicotinamide Adenine Dinucleotide Phosphate; NET, Neutrophil Extracellular Trap; NF-κB, Nuclear Factor kappa B; NLRP3, NOD-like Receptor Protein 3; NO, Nitric Oxide; NOX2, NADPH Oxidase 2; NQO1, NAD(P)H:Quinone Oxidoreductase 1; Nrf2, Nuclear factor erythroid 2-related factor 2; O_2_•^−^, Superoxide; 8-OHdG, 8-hydroxy-2′-deoxyguanosine; OMV, Outer Membrane Vesicle; ONOO−, Peroxynitrite; PAR, Protease-Activated Receptor; PKC, Protein Kinase C; PRMT, Protein Arginine Methyltransferase; PRR, Pattern Recognition Receptor; PYD, Pyrin Domain; RNS, Reactive Nitrogen Species; ROS, Reactive Oxygen Species; SOD, Superoxide Dismutase; TLR, Toll-like Receptor; TNF-α, Tumor Necrosis Factor-alpha; TRAF6, TNF Receptor-Associated Factor 6; TRIF, TIR-domain-containing adapter-inducing interferon-β; TXNIP, Thioredoxin-Interacting Protein; VCAM-1, Vascular Cell Adhesion Molecule-1; VSMC, Vascular Smooth Muscle Cell; 4-HNE, 4-Hydroxynonenal.
